# No differences in cost-effectiveness and short-term functional outcomes between cemented and uncemented total knee arthroplasty

**DOI:** 10.1186/s12891-020-03477-x

**Published:** 2020-07-09

**Authors:** R. Rassir, P. A. Nolte, J. C. T. van der Lugt, R. G. H. H. Nelissen, I. N. Sierevelt, W. C. Verra

**Affiliations:** 1 Department of Orthopaedic Surgery, Spaarne Hospital, Spaarnepoort 1, 2134 TM Hoofddorp, the Netherlands; 2 Department of Orthopaedic Surgery, Haga Hospital, The Hague, the Netherlands; 3 Department of Orthopaedics, Leiden University Medical Center, Leiden, the Netherlands; 4 Spaarne Academy, Department of Epidemiology, Spaarne Hospital, Hoofddorp, the Netherlands; 5 Department of Orthopaedic Surgery, Medisch Spectrum Twente, Enschede, the Netherlands

**Keywords:** Total knee Arthroplasty, Cemented, Uncemented, Costs, Cost-effectiveness, Quality adjusted life years

## Abstract

**Background:**

There is an ongoing debate regarding optimal fixation of total knee arthroplasty (TKA), however cost has not been addressed as profoundly. Therefore, the current study primarily aimed to compare costs and cost-effectiveness 1 year after cemented or uncemented TKA. A secondary objective was to compare short-term functional outcomes between both groups.

**Methods:**

A posthoc prospective observational multicenter cohort study of 60 cemented and 50 uncemented Low Contact Stress (LCS) knee systems. Outcome was evaluated using the EuroQol5D-3 L (EQ5D) index, in order to calculate quality adjusted life years (QALYs). Total costs were calculated considering direct costs within the hospital setting (inpatient cost) as well as direct and indirect costs outside the hospital. Cost-effectiveness (total costs per QALY), Oxford Knee Score (OKS) and Numeric Rating Scale (NRS) were compared between cemented and uncemented cases at 1 year after surgery. HealthBASKET project, a micro-costing approach, represents the Dutch costs and situation and was used to calculate hospital stay. (In) direct costs outside the healthcare (medical cost and productivity cost) were determined using two validated questionnaires.

**Results:**

Median costs per QALY were similar between cemented and uncemented TKA patients (€16,269 and €17,727 respectively; *p* = 0.50). Median OKS (44 and 42; *p* = 0.79), EQ5D (0.88 and 0.90; *p* = 0.82) and NRS for pain (1.0 and 1.0; *p* = 0.48) and satisfaction (9.0 and 9.0; *p* = 0.15) were also comparable between both groups.

**Conclusion:**

For this type of knee implant (LCS), inpatient hospital costs and costs after hospitalization were comparable between groups.

## Background

Total knee arthroplasty (TKA) has grown to one of the most frequently performed surgeries performed worldwide [1]. Even though the 2018 Dutch Arthroplasty Register report showed 93.2% of total knee implants in the Netherlands were cemented, there is an ongoing debate among knee surgeons concerning uncemented fixation [2]. Modern uncemented implants have a porous coating, with or without a calcium phosphate (or even tantalum) coating. The latter is supposed to enhance biological ingrowth of the implant. The uncemented method provides several potential benefits, including preservation of bone stock, ease of revision, and a more durable fixation at long-term (i.e. absence of cement-implant interlock breakage and wear due to cement particles) [3–6].

However, high quality evidence comparing cemented and uncemented fixation has thus far failed to show superiority of one technique over the other due to limited sample sizes and heterogeneity in implant design [7, 8]. Although of utmost importance for surgeons and both public and private payers, cost differences are often overlooked when comparing both methods. Modern uncemented implants are often more expensive than their cemented counterparts, but prolonged duration of surgery and additional equipment (cement and mixing equipment) are inherent to cemented TKA. A recent analysis by Lawrie et al. found no differences between both modes of fixation when comparing procedural costs of publicly available data on prices of cement and implants in the United States and time of surgery [9]. In contrast, another cost comparison between both fixation methods resulted in higher inpatient costs of uncemented fixation but a shorter length of stay and higher odds of being discharged home [10]. These conflicting results, urged us to evaluate whether both direct and indirect in-patient and out-patient costs were higher in uncemented TKA patients.

A comprehensive comparison of cemented and uncemented TKA patients with respect to costs and cost-effectiveness beyond the scope of surgery and in hospital admission is lacking. Therefore, the aim was to evaluate cost-effectiveness of both TKA fixation methods in patients within the first year of surgery. The secondary objective was to compare patient reported outcome measures (PROMs) regarding knee function and pain between both techniques in TKA patients.

## Methods

### Patients

A post-hoc prospective multicenter observational cohort study of TKA patients with a cemented or an uncemented Low Contact Stress (LCS; DePuy Synthes, Warsaw, IN) knee system was done. Patients were selected from a larger cohort of 466 TKA patients (FIRST trial). This was a randomized controlled trial investigating the effect of a topical fibrin sealant on knee function 6 weeks after TKA [11]. All participants were treated according to the same surgical study protocol and were treated with multimodal analgesia in pursuit of fast rehabilitation. Data was obtained from 2 participating institutions, using either the cemented (Haga Hospital, *n* = 91) or uncemented (Spaarne Hospital, *n* = 69) version of the Low Contact Stress (LCS) knee system (DePuy Synthes, Warsaw, IN). All potential participants of the follow-up study received a letter with information about the study. Hereafter, written informed consent was obtained and all questionnaires were sent by mail. Non-responders were contacted by phone after 1–2 weeks. Fifty patients were lost of follow-up, due to insufficient baseline data (*n* = 4) and no response or unwillingness to participate in the current follow up study (*n* = 46). Thus, 110 patients, with 110 TKA (60 cemented and 50 uncemented TKA) were available and included. Data were collected using Castor data management software (Castor EDC, Amsterdam, the Netherlands). The current follow-up study was approved by the institutional review board of both participating centres (METC-ZWH 17–050; ACLU 2017.0038).

### Outcome measurements

The primary outcome of the current study was cost-effectiveness of the TKA procedures with the 1 year follow-up mark as an end-point. Quality adjusted life years (QALY) were calculated for each case using the EuroQol 5D-3L (EQ5D-3L) utility index (U). The latter was calculated: *QALY* = *U* ∗ *follow up time in years* [12, 13]. Total costs (in €, based on 2013 catalogue prices) per QALY were then calculated and compared between the cemented and uncemented version of the LCS TKA. The analysis was conducted from the societal perspective considering direct costs within the healthcare system and both direct and indirect costs outside the healthcare system. Table 1 provides an overview of included costs in the analysis. Difference in catalogue prices of both implants, cement (Palacos R + G [2 × 40], Heraeus Medical GmbH, Wehrheim, Germany) and mixing equipment, operating theatre costs and total hospital stay were considered direct costs within the healthcare system. Operating theatre costs were calculated using prices per minute derived from the HealthBASKET project, which were determined using a micro costing approach and represent the Dutch costs and situation [16]. Costs of hospital stay were calculated using Dutch reference prices [14, 15]. (In)direct costs outside the healthcare system were determined using two validated comprehensive questionnaires; the institute for Medical Technology Assessment productivity cost questionnaire (iMTA-PCQ) and medical cost questionnaire (iMTA-MCQ) [18]. The iMTA-PCQ objectifies productivity losses (of both paid and unpaid work), absenteeism and presenteeism [14]. The friction cost method was applied for determination of health costs subsequent to productivity losses, using a friction period of 85 calendar days [14, 15, 19]. The iMTA-MCQ evaluates consumption of healthcare by including questions related to contacts with healthcare providers and expenditure of medication. Medical consumption costs were then calculated using the costing tool for Dutch reference prices and pharmacy purchase prices for medication in the Netherlands [14, 15, 20]. In accordance with the manual, adjustments were made in the contents of the iMTA-MCQ to exclude healthcare consumption irrelevant to TKA, a summary of which is included in Table 1. Furthermore, the recall-period for both questionnaires was adjusted to 1 year to comprehend the total postoperative period.

**Table 1:** Included costs in the current analysis, based on the costing tool for Dutch reference prices in 2013 [14, 15]

Cost category (unit)	Price per unit (€, 2013)	
	Cemented	Uncemented
Price difference implants (Δ)	–	650
Cement and mixing equipment (unit)	193	–
Theatre time (minutes) ^a^	6,35	
Total hospital stay (day)	405	
iMTA-PCQ		
Absenteeism (working hour)	34,75	
Presenteeism (working hour)	34,75	
Productivity losses of unpaid work (hour)	14	
iMTA-MCQ		
General practice (visit)	33	
Physical therapy (visit)	33	
Occupational therapy (visit)	33	
Occupational physician (visit)	81	
Domestic help (day)	21,50	
Home nursing (day)	73	
Analgesics ^b^	Variable	
Emergency department visits (visit)	259	
Hospital admission (days)	405	
Nursing facility admission (days)	168	

^a^ Derived from the HealthBASKET project [16]

^b^ Analgesics include acetaminophen, non-steroidal anti-inflammatory drugs and opioids. Costs are calculated using normalized Dutch pharmacology costs [17]

Secondary outcome was knee pain and function 1 year after index surgery, objectified using validated patient reported outcome measures (PROM’s). The Oxford Knee Score (OKS) is a short, practical and reliable questionnaire that assesses knee function and pain [21, 22]. Scores calculated range from 0 (worst outcome) to 48 (best outcome). Patients were invited to complete the OKS before and 1 year after surgery. Pain and satisfaction 1 year after surgery were additionally assessed using a Numeric Rating Scale (NRS). Pain at rest (NRS-PR) and during activity (NRS-PA) ranged from 0 (no pain) to 10 (worst pain). Satisfaction (NRS-S) ranged from 0 (worst outcome) to 10 (best outcome). Quality of life was reported using the previously mentioned EQ5D-3L utility index, supplemented with a visual analogue scale (EQ5D%), before and 1 year after surgery [12].

### Statistical analysis

SPSS Version 25.0 (IBM Corp; Armonk, NY) was used for statistical analysis. Baseline variables were expressed as means and standard deviations (SD) or medians and interquartile ranges (IQR) for normal and non-normally distributed data, respectively. A 2-tailed independent samples students T-test (or Mann Whitney U test where appropriate) was performed to assess differences in absolute costs, cost-effectiveness (costs per QALY) and 1 year postoperative PROM’s between both groups. Missing cost data were replaced using multiple imputation, based on available baseline and follow-up data. *P*-values < 0.05 were considered statistically significant.

## Results

The 110 evaluated TKA patients (61 females) had a mean age of 65 years SD 9.0 years. Of this group, 60 had cemented TKA and 50 uncemented TKA (Table 2). A lost to follow-up analysis revealed that the excluded group was significantly older at the time of surgery than the included cohort (mean age 68 [SD 10] versus 64 [SD 9]; *p* = 0.01). Other baseline characteristics, baseline PROMs and baseline knee function were similar between in- and excluded patients. Comparison of baseline and clinical characteristics between both included groups revealed a lower preoperative knee flexion in the uncemented group (110 versus 125 degrees, *p* < 0.01) and a significantly longer total hospital stay in the cemented cohort (IQR 3–4 versus 2–3, *p* < 0.01). Further baseline characteristics were equal between groups and are summarized in Table 2.

**Table 2:** Baseline characteristics and preoperative PROMs

	Cemented	Uncemented	Overall	*p*-value
Knees, n	60	50	110	–
Age, mean (SD)	66 (9)	63 (8)	65 (9)	0.18 ^a^
BMI, mean (SD)	28.3 (5.9)	28.9 (4.2)	28.5 (5.1)	0.54 ^a^
Gender, n (%)				0.62
Female	29 (58.0)	32 (53.3)	61 (55.5)	
Male	21 (42.0)	28 (46.7)	49 (44.5)	
ASA classification, n (%)				0.72
I	9 (18.0)	7 (13.5)	16 (15.7)	
II	40 (80.0)	43 (82.7)	83 (81.4)	
III	1 (2.0)	2 (3.8)	3 (2.9)	
Smoking, n (%)				0.42
Yes	6 (12.0)	10 (17.5)	16 (15.0)	
No	44 (88.0)	47 (82.5)	91 (85.0)	
Knee flexion (°), median (IQR)	125.0 (102.0; 140.0)	110.0 (100.0; 120.0)	120.0 (100.0; 126.0)	**< 0.01**^**b**^
Operation time (minutes), mean (SD)	63.0 (17.0)	56.9 (10.5)	60.4 (14.7)	**0.02**^**a**^
Total hospital stay (days), median (IQR)	3.0 (3.0; 4.0)	3.0 (2.0; 3.0)	3.0 (3.0; 4.0)	**< 0.01**^**b**^
PROMs				
OKS, mean (SD)	28 (7)	26 (8)	27 (8)	0.22 ^a^
EQ5D, median (IQR)	0.78 (0.66; 0.81)	0.81 (0.63; 0.81)	0.81 (0.65; 0.81)	0.81 ^b^
EQ5D%, median (IQR)	80.0 (70.0; 90.0)	76.0 (65.0; 90.0)	78.0 (69.0; 90.0)	0.37 ^b^

Numbers do not add up to total due to missing data. Percentages are portions of the assigned group (cemented/uncemented). *BMI* Body mass index, *SD* Standard deviation, *IQR* Interquartile range, *ASA* American Society of Anesthesiologists, *PROMs* Patient reported outcome measures

^a^ independent samples T-test ^b^ Mann Whitney U test

Overall in-patient and out-patient costs (iMTA-MCQ and iMTA-PCQ) were comparable between both cemented and uncemented TKA patients, median costs €11,131 (IQR 6192; 26,405) and €13,640 (IQR 7036; 29,859) respectively (*p* = 0.52; Table 3). Cost-effectiveness, costs per QALY, displayed a median of €16,269 (IQR 7300; 33,318) in the cemented TKA group and in the uncemented TKA group a median of €17,727 (IQR 8739; 42,696) (*p* = 0.50; Table 3). PROMs at 1 year after index surgery were comparable as well (Table 3).

**Table 3:** Comparison of costs, cost-effectiveness and PROMs between cemented and uncemented cases one year after index surgery

	Cemented	Uncemented	Total	*p*-value*
Costs per QALY (€), median (IQR)	16,269 (7300; 33,318)	17,727 (8739; 42,696)	16,958 (7960; 37,336)	0.50
Total costs (€), median (IQR)	11,131 (6192; 26,405)	13,640 (7036; 29,859)	12,400 (6529; 26,589)	0.52
OKS, median (IQR)	44 (39; 46)	42 (36; 47)	43 (37; 46)	0.79
NRS-PR, median (IQR)	0.0 (0.0; 1.0)	0.0 (0.0; 2.0)	0.0 (0.0; 1.8)	0.75
NRS-PA, median (IQR)	1.0 (0.0; 2.0)	1.0 (0.0; 2.3)	1.0 (0.0; 2.0)	0.48
NRS-S, median (IQR)	9.0 (6.8; 10.0)	9.0 (7.8; 10.0)	9.0 (7.0; 10.0)	0.15
EQ5D, median (IQR)	0.88 (0.81; 1.00)	0.90 (0.78; 1.00)	0.89 (0.81; 1.00)	0.82
EQ5D%, median (IQR)	79.5 (60.0; 90.0)	80.9 (70.9; 96.0)	80.0 (62.4; 93.0)	0.17

*PROMs* Patient reported outcome measures, *IQR* Interquartile range, *OKS* Oxford Knee Score, *NRS* Numeric Rating Scale

* All differences are assessed with Mann Whitney U test

## Discussion

The current study demonstrated, in a post-hoc analysis using both cemented and uncemented components of the same TKA design, comparable average costs and cost-effectiveness per patient up to 1 year after the index surgery. Even though we found higher median costs per QALY in the uncemented group, interquartile ranges were wide and differences were therefore statistically insignificant (*p* = 0.50). Furthermore, PROMs (pain scores and knee function) were comparable between both groups at 1 year follow-up. The current analysis is the first cost and cost-effectiveness comparison of cemented and uncemented TKA from a European market perspective (Table 3). This is in accordance with the previous analysis by Lawrie et al. but in contrast with the study performed by Gwam et al. [9, 10]. This discrepancy is most likely explained by the latter analysis being a large database analysis focused on charges of hospital admission, not accounting for the lower operative time in uncemented TKA [10]. Another important note is the fact that implant prices are influenced by economic climate and therefore fluctuate constantly. Prices of uncemented implants are decreasing in the last years and, as a consequence, are almost the same as the prices for cemented implants nowadays (unpublished industry data). For that matter, the costs of implants at surgery are only a minor part of the overall costs related to hospital admission, use of physiotherapy, analgesics and ultimately survival of the implant (i.e. prevention of loosening). Data from national registries at 10 years show a mean revision rate with 95% confidence interval of the most implanted uncemented TKA of 5.3% (4.6–5.9) in the Netherlands (LCS; Dutch Arthroplasty Register), 3.4% (3.1–3.8) in the UK and Wales (LCS; National Joint Registry) and 4.1% (3.7–4.5) in Australia (Triathlon; Australian Orthopaedic Association National Joint Replacement Registry) [2, 23, 24]. Whilst 10 year revision rate of the most implanted cemented TKA in these three countries is 6.3% (5.9–6.6; Genesis II), 2.5% (2.4–2.5; PFC Sigma) and 3.8% (3.5–4.1; Triathlon) respectively [2, 23, 24]. As most revision rates in these registers are comparable between cemented and uncemented TKA, the decision should be made based on the preference and training of the knee surgeon.

We found no differences in knee pain and function 1 year after cemented or uncemented TKA. Previous comparisons tend to show similar results, even after longer follow-up [8]. A study by Park and Kim compared bilateral TKA using cemented fixation on one side and uncemented fixation on the other [5]. They reported similar results in terms of knee function and pain after a mean follow-up of 13.6 years, which suggests the findings of the current study most likely to be durable [5].

Some limitations have to be discussed. This study is a post-hoc analysis of TKA patients participating in a randomized controlled trial, but is the first to report on cost-effectiveness instead of just costs in TKA patients and has a Dutch market perspective (i.e. private health care insurance). Firstly, the non-responders could have an effect on our results due to the possibility of selection bias. However, we performed a comparison between both groups which revealed age at surgery as the only characteristic to be different between in- and excluded patients. Secondly, recall-bias may be introduced in the iMTA questionnaires due to the extent of the recall-period employed (1 year instead of 3 months). Moreover, heterogeneity of the included population due to different institutions both cohorts are derived from may have an effect on total hospital stay (due to differences in physiotherapists and protocols, nursing staff attitudes, consultant attitudes) and surgical time (due to differences in training and experience of the surgeons in both institutions). Furthermore, preoperative knee flexion is a known predictor of patient satisfaction and was lower in the uncemented group, which may introduce confounding in the interpretation of PROMs [25]. Since the current findings are strongly supported by the existing literature [8], we expect the chance of confounding to be low. Another possible limitation is the inability of a sample size calculation due to the retrospective nature, which introduces the possibility of the current study to be underpowered. However, as the current findings are supported by the literature [9] we expect this possibility to be minimal. Lastly, revisions may attribute to additional costs and were not taken into account. Due to previous comparisons showing similar revision rates [7, 8], we expect this additional expense to be evened out between groups.

As the burden of TKA increases, health care payers and orthopaedic surgeons are forced to investigate efficient use of resources [26]. Even more, orthopaedic surgeons should base their decisions on evidence, no innovation without evaluation [27]. One means to accomplish this is to evaluate knee systems with micromotion analysis of the implant within the bone, since early migration is associated with failure at 5 and 10 years [28, 29]. Due to the parallel rejuvenation of the population undergoing TKA and the development of modern implants, uncemented fixation has regained interest among arthroplasty surgeons worldwide [26]. The durable, biological fixation of uncemented implants is theorised to provide more longevity and therefore lower costs in terms of revision in younger patients [30]. Conversely, the main criticism of uncemented fixation is inferior survivorship of the tibial component in large observational studies compared to cemented implants [31–33]. Although not the optimal study design to investigate implant survival, randomised trials tend to challenge these results by showing similar survivorship regardless of fixation method [5, 7, 8, 32–34]. This discrepancy may be explained due to the fact that observational and registry studies tend to include uncemented implants with either design-related failures or without a hydroxyapatite coating, the latter has been shown superior to non-coated implants [32, 33, 35–37]. Another criticism is the often higher purchase price of uncemented implants [10, 38]. The current study confirms, however, that when investigating costs beyond just implant purchase these differences are diminished due to longer operating time and additional costs of cement and mixing equipment in cemented TKA. Comparative meta-analyses pooling data from modern (hydroxyapatite or tantalum coated) uncemented implants are lacking and might deliver evidence on the optimal mode of fixation with regards to long-term survival.

## Conclusion

Overall costs and cost-effectiveness including both in-patient and out-patient costs at 1 year after cemented and uncemented knee replacement were comparable. Cost should not be a concern associated with uncemented TKA, especially in the modern market where the additional costs compared to a cemented implant tend to become less significant. However, long-term results from registries warrant caution when using (uncoated) uncemented implants.

## Data Availability

The datasets used and/or analysed during the current study available from the corresponding author on reasonable request.

## Abbreviations

TKA: Total knee arthroplasty
LCS: Low Contact Stress
PROM: Patient reported outcome measure
QALY: Quality adjusted life year
U: Utility index
EQ5D: EuroQol5D-3L
OKS: Oxford Knee Score.
NRS: Numeric Rating Scale.
iMTA-PCQ: Institute for Medical Technology Assessment productivity cost questionnaire
iMTA-MCQ: Institute for Medical Technology Assessment medical cost questionnaire
SD: Standard deviations
IQR: Interquartile range.
